# Supplementary material for: Comparative genomics and full-length Tprk profiling of Treponema pallidum subsp. pallidum reinfection
Source: PLoS Negl Trop Dis. 2020 Apr 6;14(4):e0007921. doi: 10.1371/journal.pntd.0007921 (PMC7162541; doi:10.1371/journal.pntd.0007921)
Supplement: S1 Table — (DOCX) [file pntd.0007921.s001.docx]

**Supplemental Table 1.** PCR primers used in this study for gap filling and deep tprK profiling.

| **Name** | **Sequence (5´ –> 3´)** |
| --- | --- |
| UW-148B Repetitive Region Primers | |
| *148B_tprC-F* | CAA AGG CAA GAC GAG CAA AAC G |
| *148B_tprC-R* | CTA TTC GAA ACA TCG CCG GAA CC |
| *148B_rRNA-1-F* | GAG AAG GTG TCA TTT GGG GTT GG |
| *148B_rRNA-1-R* | CGA TAT CTT TCG TGA GTG GAC GG |
| *148B_rRNA-2-F* | GAC AAG GGG TAT AGG TTA TGG G |
| *148B_rRNA-2-R* | GAC GTT CCC GAT TAC TTC TCC C |
| *148B_tprIJ_F* | CCT TGG CAG TGC ACG TTA GCA C |
| *148B_tprIJ_R* | CAG GCA TAT CGC TTG TAT CCG C |
| UW-148B2 Repetitive Region Primers | |
| *148B2_tprC-F* | CAA AGG CAA GAC GAG CAA AAC G |
| *148B2_tprC-R* | CTA TTC GAA ACA TCG CCG GAA CC |
| *148B2_tprD-F* | CAC ATT GCT GGC CTT CGA TGC |
| *148B2_tprD-R* | CCG CGA ATG AGT GGC TCT AAC C |
| *148B2_rRNA-1-F* | CGG TTC GTC TTC CAG TTG GAG |
| *148B2_rRNA-1-R* | GAG ATC CTT TGC ACT GTC CAC TC |
| *148B2_rRNA-2-F* | GAC AAG GGG TAT AGG TTA TGG G |
| *148B2_rRNA-2-R* | GAC GTT CCC GAT TAC TTC TCC C |
| *148B2_tprIJ-F* | CTC CTC CCC GCA ATT CTT CGA C |
| *148B2_tprIJ-R* | GTA TGA ACT GGC GCA GGG TAA CTG |
| *148B2_tprK-F* | CAA CAA GCC CTC CTA AAA GGA GC |
| *148B2_tprK-R* | GTG CAG GAT CTC TTC TCG GAC C |
| *tprE/F/G* Primers | |
| *tprEFG_full-F* | GGA ACT GCA GCC GGG ATG |
| *tprEFG_full-R* | CAA GGA AAG AGC CGC ATG CTA CC |
| *tprEFG_segmentA-F* | GCG TTT GCG CTC GCA AAG G |
| *tprEFG_segmentA-R* | GCA CCA CAG CGT TGA TCC TC |
| *tprEFG_segmentB-F* | CAG CAA GTT CTT GTG TTC AAC C |
| *tprEFG_segmentB-R* | CAG GGT GGA GCA ATA CCG |
| *tprEFG_segmentC-F* | CTC GGC AAT ACC CAT GTA AGG |
| *tprEFG_segmentC-R* | CTT GAT GCC GTA ACG GCA GC |
| *tprEFG_segmentD-F* | GTG ACG GTG TAG GAG GGT C |
| *tprEFG_segmentD-R* | CGA AAT ACC TTC CCA CAC CTG |
| *arp* Primers | |
| *arp_outer-F* | GGG AAT ACG TAA GGT GTC TGC |
| *arp_outer-R* | GGT CAC GAG GGG AAG AGG |
| *arp_inner-F* | CAC ACT GTA CCG GAA TAT CGC |
| *arp_inner-R* | CGA AAC TGA TTG GGA GCT GAG |
| Pacbio Barcodes with *tprK* Gene-specific Primers | |
| *bc1001-tprK-F* | CAC ATA TCA GAG TGC GGG AAA GAA AAG AAC CAT ACA TCC |
| *bc1002-tprK-F* | ACA CAC AGA CTG TGA GGG AAA GAA AAG AAC CAT ACA TCC |
| *bc1009-tprK-R* | ACA CAC GCG AGA CAG ACG CAG TTC CGG ATT CTG |
| *bc1010-tprK-R* | ACG CGC TAT CTC AGA GCG CAG TTC CGG ATT CTG |
